# Problems with non-adherence to antipsychotic medication in Samoan new Zealanders: A literature review

**DOI:** 10.1111/j.1447-0349.2011.00801.x

**Published:** 2012-08

**Authors:** Itagia Ioasa-Martin, Laurie Jo Moore

**Affiliations:** 1 Mangere Community Health Trust (Primary Health Organisation)Auckland, New Zealand; 2 Mental Health Unit, Cairns Base HospitalCairns, Queensland, Australia

**Keywords:** adherence, compliance, health services, Pacific Island mental, Samoan traditional beliefs

## Abstract

This paper explores what is known about adherence to antipsychotic medications in general and the possible reasons for non-adherence in Samoan New Zealanders. Samoan New Zealanders are either Samoan-born immigrants or their descendents born in New Zealand. Clinicians recognize a high prevalence of non-adherence among Samoan New Zealanders. The authors hypothesize that traditional Samoan beliefs play a prominent role in problems with adherence. To investigate this hypothesis, a review of the literature on adherence in Samoan New Zealanders was undertaken. Documents from the Ministry of Health support the hypothesis. To investigate this issue, the Ministry of Health initiated a qualitative research project to examine the nature of Samoan traditional beliefs. The results of this study are summarized. No research had previously been undertaken on adherence in Samoan New Zealanders. In general, there is a lack of research on all aspects of the mental health of Pacific peoples in New Zealand. Literature reviews of adherence research consistently show that interventions that improve adherence address the beliefs, behaviours, and relationships surrounding adherence. This finding supports the author's hypothesis that traditional beliefs play an important role in the problem of adherence. Further definitive study with Samoan New Zealanders is required.

## INTRODUCTION

Antipsychotic medication is considered one of the great accomplishments of the twentieth century (Dally 2000; Koop 1996). However, many people from traditional cultures are distrustful of Western medicine. Traditional belief systems often conflict with biological knowledge about the causes and treatment of mental illness.

Psychotic disorders in Samoan New Zealanders include schizophrenia, schizoaffective disorder, mood disorders with psychotic features, and psychosis not otherwise specified. The treatment for psychotic disorders usually requires antipsychotic medication. The failure to take medication for psychotic disorders is a serious problem that leads to unnecessary suffering (Bathgate 1994; Tamasese *et al*. 1997). This paper focuses on the problem of adherence because much better treatment should be available to Samoan New Zealand families with consumers who suffer from psychotic disorders.

Samoan New Zealanders consist of a mixture of 50% Samoan-born immigrants and 50% New Zealand-born residents. These two groups have different cultural, social, and economic dynamics, but within their families, they are still bound by traditional Samoan beliefs to different degrees, depending on the influence of their extended families.

The term ‘Pacific peoples’ refers to the population of New Zealanders of South Pacific Island origin, which includes at least 22 different ethnic groups (Tukuitonga & Finau 1997) and many people of mixed ethnicity (Bedford & Didham 2001). Even though there are similarities, each ethnic group has distinct differences in language, social structure and history (Tukuitonga & Finau 1997). Samoa is the largest of the Pacific Islands (Bedford & Didham 2001) and Samoans represent more than 50% of the Pacific peoples' population in New Zealand. Other Pacific ethnic groups in decreasing order of prevalence include Cook Island Maori, Tongan, Niuean, Fijian, Tokelauan, and Tuualvan. Approximately 93% of Pacific peoples live on the North Island and approximately 67% live in Auckland (Wright & Hornblow 2008).

Samoan elders say that mental illness did not exist in Samoa, or, if it existed, it was very rare. They reject the Western concept that mental illness is biological (Tamasese *et al*. 1997; 2005). They do not understand terms or concepts used to describe diagnosis or treatment (Coombs *et al*. 2003), or the value of medication (Arthur 2002).

Samoan people began migrating to New Zealand over 100 years ago (Finau & Tukuitonga 2000) and they now account for 6.9% of the population in New Zealand (Wright & Hornblow 2008). Moving form Samoa to New Zealand has been challenging for many Samoan people.

## MENTAL HEALTH TREATMENT FOR SAMOAN NEW ZEALANDERS: PACIFIC ISLAND MENTAL HEALTH SERVICES

The Treaty of Waitangi signed by the Chiefs of the Confederation of United Tribes of New Zealand and the English Crown in 1840 paved the way for the development of a bicultural society that extended equal rights to Maori for protection, participation, and partnership, both in government and health care (King 2003). The right to culture-specific care has been extended to Pacific peoples, including Samoan New Zealanders.

In 1997, as part of a national initiative to improve mental health care for Pacific peoples, the Pacific Island Mental Health Services (PIMHS) were established (Ministry of Health 1997). These services provide consultation and direct services that are either regional or area-specific. PIMHS try to deliver care that is respectful of the culture of Pacific peoples. They employ as many Pacific peoples as possible, but because the number of Pacific people in mental health is limited, they also employ cultural support workers. A model of care for Pacific people was developed similar to the Maori model of care. Unfortunately, no research has been done to study the efficacy of these interventions.

### Method

A literature review was conducted using search words such as ‘non-adherence’ or ‘non-compliance’ alone and in combination with ‘Samoan People in New Zealand’, ‘Pacific Island People in New Zealand’, ‘Pacific peoples’‘cultural beliefs and values’, ‘mental health’, ‘psychiatric’, and ‘nursing’. Searches were conducted in all databases available on the Auckland District Health Board intranet, including Medline, Ovid, and Psych-Info, and library indexes of medical and nursing journals. All relevant documents from the Ministry of Health were reviewed. Some studies were related more to adherence and others to Pacific or Samoan people, but few covered these two areas. Both the quantity and quality of research relating to the subject of interest were severely lacking.

In reviewing adherence, only studies that met the standard for properly conducted randomized controlled trials (RCT) with doubled-blinded methodology were included. One qualitative study served as the primary source on Samoan traditional beliefs.

### Definitions

When a consumer stops taking medication prescribed by a medical professional for the treatment of a certain disorder, this behaviour is described as ‘non-compliance’ or ‘non-adherence’ (Dodds *et al*. 2000; Haynes 2002). ‘Compliance’ is understood as the extent to which a consumer's behaviour in terms of taking medication coincides with medical advice (Dodds *et al*. 2000). ‘Non-compliance’ is the extent to which a consumer's behaviour in taking medication does not coincide with medical advice. The word ‘compliance’ suggests that the consumers should accept the instructions of mental health professionals. The term ‘adherence’ suggests a more active role for the consumer, who makes a choice in whether or not to follow medical instructions. ‘Non-adherence’ is defined as the decision of the consumer not to take prescribed medication, while not entirely rejecting other biomedical advice (Dodds *et al*. 2000). The use of the term ‘adherence’ is preferable, but, in the present study, the term ‘compliance’ will be used where it applies to research investigations and interventions that were developed using this term.

Traditional Samoan beliefs are the beliefs of Samoan elders who were either Samoan-born or learned traditional Samoan beliefs from Samoan family members.

### Literature review of adherence and the effectiveness of antipsychotics

Despite the discovery of antipsychotic mediations, many people with psychotic illnesses remain unwell (Dixon *et al*. 1995). Non-adherence is recognized as a major problem (Coombs *et al*. 2003). Non-adherence is highly prevalent among Samoan New Zealanders with psychotic disorders and it is a major cause of relapse and hospital readmission (Bathgate 1994; Tamasese *et al*. 1997).

Within 1 year of beginning treatment, 50% of consumers with psychotic disorders stop taking medication, and 75% stop within 2 years (Weiden & Olfson 1995). Similar rates of non-adherence are observed in people suffering from a wide range of physical illnesses (Haynes 2002).

Forty-four RCT comparing antipsychotics with placebo show that antipsychotics are effective in the treatment of psychotic disorders 72% of the time (Dixon *et al*. 1995). In the community, where consumers may not adhere to medication, however, studies show an effectiveness rate of 50% (Dixon *et al*. 1995). Theoretically, improving adherence should improve efficacy (Kissling 1994; Miller *et al*. 1997) and studies show that addressing non-adherence leads to better outcomes (Blackwell 1996; Gray *et al*. 2002; Kemp *et al*. 1998).

### Overview of factors influencing non-adherence

Several meta-analyses studies show that non-adherence is a complex behaviour affected by a wide variety of factors involving the individual, the environment, and the availability of health-care services (Happell *et al*. 2002; Lacro *et al*. 2002; Miller *et al*. 1997; Zygmunt *et al*. 2002). Consumer-related risk factors include poor insight, negative beliefs about medication, previous non-adherence, shorter duration of illness, and coexisting substance misuse. Environmental risk factors predictive of non-adherence include a poorer therapeutic relationship, less outpatient contact, inadequate discharge planning, and a poor aftercare environment. Treatment-related factors, such as the type of medication, the route of administration, the regimen complexity, higher dose, and the side effects of medication do not consistently predict adherence. A numbers of factors that one might expect to be related to non-adherence, such as age, gender, ethnicity, marital status, education level, involvement of family, severity of symptoms, presence of mood symptoms, and neuro-cognitive impairment have not been found to be significant predictors of non-adherence in the majority of studies that examined them.

There are inconclusive and paradoxical findings about negative side effects, such as weight gain, akathisia, extra-pyramidal symptoms, and sexual dysfunction. Although one might expect these factors to increase non-adherence, improvement in the doctor–consumer relationship and improved education have a positive influence that outweighs the consequences of side effects (McCann & Clark 2003).

People from traditional cultures often expect medications to work quickly and they stop taking medication if they do not experience immediate relief (Burroughs 2003; Westermeyer 1985; Westermeyer 1989). Samoan people stop taking medication when they feel they have recovered (Tamasese *et al*. 1997; 2005).

### Adherence-enhancing interventions

Approaches that improve adherence include educational, behavioural, emotional or motivational consumer and family focused interventions and those that target the behaviour of doctors, pharmacists, or nurses (Roter *et al*. 1998)

Ethnic populations require the provision of safe and culturally appropriate mental health services (Flinders University School of Nursing and Midwifery 2003) that is offered to consumers in their own language (Berg 2003). Community interventions that improve access to mental health care improve adherence (Fleischhacker 2002). The PIMHS endeavours to address all these issues for Samoan New Zealanders.

In the PIMHS, Samoan nurses play a critical role in affirming the Samoan culture and providing education about mental illness and the benefit of medication. Nurses provide education, advocacy, reassurance, family support, and liaison services, but they are not educated about antipsychotic medication in a consistent way and they often feel a conflict between their role as educators and as advocates (Happell *et al*. 2002). In their liaison role, nurses influence the access to mental health services and need to pay special attention to establishing good rapport with consumers and caregivers, especially mothers. Complex phone systems need to be avoided (Happell *et al*. 2002). Workloads that are too high hamper nurses' ability to respond to the needs of consumers and families (Happell *et al*. 2002).

The expansion and further development of PIMHS has been recognized and called for by Pacific peoples' leaders and organizations in New Zealand (Ministry of Pacific Island Affairs 1999).

A systematic review of 13 of 39 medication adherence-enhancing studies between 1980 and 2000 helped to clarify effective interventions (Zygmunt *et al*. 2002). Individual and family psycho-education improves knowledge but not adherence. Combining education with behavioural or motivational approaches or accompanying supportive services improves adherence (Gray *et al*. 2002). When consumers collaborate in treatment decisions, consumer confidence in medication improves and more trusting relationships are formed (Dodds *et al*. 2000). Assertive case management models have only modest efficacy. Short-term adherence can be achieved relatively easily using education and reminders. For long-term adherence even the most effective combination of interventions does not lead to large improvements in adherence. Furthermore, efforts to improve low adherence must be maintained as long as the treatment is needed. The simplest and most effective intervention is to telephone consumers who miss appointments and make every effort to keep them in care (Haynes 2002). Individualized behaviour-tailoring interventions, including collaborative definition of early warning signs, relapse prevention, and comprehensive management plans, are generally successful (Dodds *et al*. 2000; Zygmunt *et al*. 2002). Cognitive behavioural therapy (CBT) that targets attitudes toward medication (Lecompte & Pelc 1996) has been incorporated into a structured intervention called ‘compliance therapy’ that has been shown to be effective (Hayward & Chan 1995; Kemp *et al*. 1996; Kemp *et al*. 1998; Zygmunt *et al*. 2002). Specific targeted interventions that address medication non-adherence are more effective than broad-based programs covering a wide range of problem areas (Zygmunt *et al*. 2002).

Compliance therapy is a brief, pragmatic, manual-based intervention that consists of four to five sessions plus booster sessions. It involves using CBT, motivational interviewing, and psycho-education, which target the therapeutic relationship, the consumer's ambivalence about taking medication, and the consumer's insight. The principles are: working collaboratively, emphasizing personal choice and responsibility, and focusing on consumers' beliefs and concerns about treatment (Kemp *et al*. 1997). Compliance therapy has been tailored for psychiatrists, psychologists, and nurses. Those forms tailored for psychiatrists, psychologists, and nurses have been successfully combined (Zygmunt *et al*. 2002).

Effective adherence interventions address the thoughts, beliefs, feelings, behaviours, and relationships surrounding adherence. These findings are consistent with the hypothesis that traditional Samoan beliefs play a significant role in adherence problems in Samoan New Zealanders with psychotic disorders.

The next section presents an overview of Samoan elders' views.

### Stressors specific to Samoan New Zealanders

In 1997, the Ministry of Health commissioned a qualitative research project with Samoan elders to explore their views on the causes of mental illness (Tamasese *et al*. 1997; 2005). The elders felt that acculturation and socioeconomic stressors were the primary cause of mental illness in Samoan New Zealanders and that these factors were not given proper consideration in their treatment.

Among ethnic groups, Pacific peoples have the lowest income (Miller *et al*. 1997; Ministry of Pacific Island Affairs 1999) and the highest rate of unemployment in New Zealand (Ministry of Pacific Island Affairs 1999). In keeping with the elders' beliefs, it has been shown that poverty doubles the risk of developing mental illness, decreases the life span, and increases the rate of death from all causes (Moore 2000). Poverty leads to problems with transportation and buying medication, which interfere with effective treatment of Samoan New Zealanders (Esera 2001; Miller *et al*. 1997; Ministry of Health 2002).

Samoan New Zealanders experience intense shame when a family member attends mental health services or takes psychiatric medications (Malo 2000), and families actively prevent consumers from taking medications and attending appointments.

### Traditional Samoan beliefs

#### Alienation and breakdown of Samoan culture

Samoan people think of their culture as an essential and enduring part of their wellness and identity. If a Samoan person becomes unwell for any reason, that person requires the support of the Samoan community to regain wellness. Alienation from Samoan culture is considered a major cause of being unwell (Tamasese *et al*. 1997; 2005).

The Samoan family is the foundation of Samoan identity and wellness. From a traditional perspective, an individual does not exist separately from the family. When a Samoan person is unable to maintain connection with family and Samoan society or when they are unable to fulfil familial responsibilities, their self has no cultural identity, no sense of belonging, and no purpose outside the cultural context and the collective connections that give life meaning. Factors that disrupt the traditional paradigm of wellness include the absence of interaction between extended families and the lack of traditional structures of support (Tamasese *et al*. 1997; 2005).

#### Maledictory invocations or curses and spirit possession

Relational arrangements are the linchpin of Samoan protocols and etiquette that define appropriate behaviour and protect people's wellbeing. Samoan protocols, etiquette, and relational arrangements are considered *tapu*, which means ‘that which is forbidden to the ordinary’, and *sa*, meaning *‘s*acred’. Breaches of tapu and sa are grave infractions that may result in maledictory invocations or curses that cause mental illness or even death. To be outside relational arrangements is to be incorrectly placed in terms of identity and belonging, such that a person who desecrates the sacredness of parents, protocols, etiquette, and traditional nobility is considered crazy, stupid, or mentally unwell.

Curses can be placed by parents or people with authority like chiefs or heads of the family. Curses may be carried forward into generations and it is for this reason, the elders say, that mental illness runs in families. Curses may be brought on people for failure to provide for their family, bringing shame to the family, or failure to comply with the wishes of a dying person. Curses condemn the offender to a life of wandering or a life without purpose, and they are truly an anathema to any Samoan person.

The Samoan word that most closely describes mental illness means ‘spiritual illness’ or ‘possession by the spirit’ (Tamasese *et al*. 1997; 2005). Although most Samoan New Zealanders are Christians and believe in God, they continue to believe in their traditional gods and perceive the presence of spirits and gods in their everyday life (Tamasese *et al*. 1997; 2005). Samoans believe that mental illness is either a punishment by God or Samoan gods (Logologo 1996), or the result of possession by an evil spirits for the sins of the afflicted individual or some family member (Gluckman 1997; Kinloch 1985; Malo 2000). Ghosts are believed to be the bearers of curses (Logologo 1996) or the possession by evil spirits (Tamasese *et al*. 1997; 2005).

A person may be struck and possessed by a spirit, for example, for showing disrespect in a place where that spirit is the guardian. If the person is to be healed, a traditional healer must identify the offending spirit by a mark or imprint left on the body (Tamasese *et al*. 1997; 2005).

Traditional healers are widely accepted by Samoan New Zealanders and traditional practices include herbal remedies, massage, and the removal of evil spirits (Malo 2000).

Effective and culturally safe treatment requires the involvement of the family in almost all clinical interactions.

## DISCUSSION, CONCLUSION AND IMPLICATIONS

Acculturation and socioeconomic stressors are understood by Samoan elders to be the major causes of mental unwellness in Samoan New Zealanders. Samoan elders do not believe that mental illness is biological. They believe psychological disruption is caused by alienation from Samoan culture, disturbances of familial relationships, failure to meet family financial obligations, and straying from traditional values. Samoan elders believe that mental illness is due to spirit possession, or spiritual punishment by the imposition of curses for breaching sacred protocols, etiquette, or relational arrangements.

There is reason to believe that traditional Samoan beliefs are relevant to problems with adherence among Samoan New Zealanders. Compliance therapy and individualized behavioural interventions need to be developed to incorporate the traditional belief system of the Samoan New Zealanders into the PIMHS.

Extensive educational initiatives are needed to teach Samoan New Zealanders about mental illness and medication treatment. Adequate funding is required to expand culture-specific services for Samoan New Zealanders and to support research into interventions that incorporate the traditional belief systems of Samoan New Zealanders.
